# The real-world patient experience of fingolimod and dimethyl fumarate for multiple sclerosis

**DOI:** 10.1186/s13104-016-2243-8

**Published:** 2016-09-07

**Authors:** Paul Wicks, Lawrence Rasouliyan, Bo Katic, Beenish Nafees, Emuella Flood, Rahul Sasané

**Affiliations:** 1 PatientsLikeMe, 160 Second Street, Cambridge, MA 02142 USA; 2 ICON Plc, Medical Affairs Statistical Analysis, Torre Diagonal Mar, Josep Pla, 2, Planta 11, Módulo A1, 08019 Barcelona, Spain; 3 ICON Plc, Clinical Outcomes Assessments, 820 W Diamond Ave Ste 100, Gaithersburg, MD 20878 USA; 4 Novartis Pharmaceuticals Corporation, 1 Health Plaza, East Hanover, NJ 07936 USA

**Keywords:** Relapsing-remitting multiple sclerosis, Fingolimod, Dimethyl fumarate, Treatment discontinuation, Patient satisfaction, Side effects

## Abstract

**Background:**

Oral disease-modifying therapies offer equivalent or superior efficacy and greater convenience versus injectable options.

**Objectives:**

To compare patient-reported experiences of fingolimod and dimethyl fumarate.

**Methods:**

Adult relapsing-remitting multiple sclerosis patients treated with fingolimod or dimethyl fumarate were recruited from an online patient community and completed an online survey about treatment side effects, discontinuation, and satisfaction.

**Results:**

281 patients in four groups completed the survey: currently receiving fingolimod (CF, N = 61), currently receiving dimethyl fumarate (CDMF, N = 129), discontinued fingolimod (DF, N = 32) and discontinued dimethyl fumarate (DDMF, N = 59). Reasons for treatment switch were to take oral treatment (CF: 63.3 %, CDMF: 61.8 %), side effects of prior medication (CF: 67.3 %, CDMF: 44.1 %) and lack of effectiveness of prior medication (CF: 38.8 %, CDMF: 31.4 %). Main reasons for discontinuation were side effects (DF: 46.9 %, DDMF: 67.8 %) and lack of effectiveness (DF: 25.0 %, DDMF: 15.3 %). CDMF patients had an increased risk of abdominal pain, flushing, diarrhea, and nausea. Treatment satisfaction was highest among CF patients followed by CDMF, DF, and then DDMF patients.

**Conclusions:**

Discontinuation was driven by experience of side effects. Patients currently taking dimethyl fumarate were more likely to experience a side effect versus patients currently taking fingolimod. Examination of the relationship between tolerability and adherence/persistence is needed.

## Background

Multiple sclerosis (MS) is a chronic inflammatory demyelinating condition of unknown etiology. It is one of the most common diseases of the central nervous system, affecting more than 2.3 million people worldwide [1]. Approximately 90 % of patients have the relapsing-remitting form of MS (RRMS) initially [1]. With no known cure, disease-modifying therapies (DMTs) aim to prevent relapses, as well as delay the accumulation of disease burden and disability [2]. From 1993 until recently, DMTs approved by the US Food and Drug Administration (FDA) were in injectable form, but these have been associated with sub-optimal adherence, and increased risk of relapse and accumulation of disease burden [3]. Reasons for non-adherence include injection-related factors, as well as side effects of the medications themselves [4–6].

Fingolimod (Gilenya^®^, Novartis Pharmaceuticals Corporation) [7], an oral DMT approved by the FDA in 2010, has shown to have superior efficacy to a comparator injectable DMT [8] and there is evidence of higher adherence and persistence, thought to be attributable in part to the improved tolerability profile as well as the mode of administration [2, 5]. Although fingolimod is well-tolerated, it requires monitoring for 6 h after administering the first dose. Dimethyl fumarate (Tecfidera^®^, Biogen) [9], approved by the FDA in 2013, is another oral DMT with favorable efficacy to injectable DMTs [10]. Unlike fingolimod, dimethyl fumarate requires no monitoring, but reports suggest it has been challenging for some patients to tolerate, particularly within the first 60 days of initiating treatment [11].

Tolerability of fingolimod and dimethyl fumarate has been assessed in clinical trials and retrospective studies [2, 5, 12], rather than in less-controlled, real-world population studies. This study was designed to explore the real-world, patient-reported experiences associated with initiating treatment with either fingolimod or dimethyl fumarate. The specific objectives of this patient-reported study were to compare the side effect experiences of patients on fingolimod and dimethyl fumarate, to compare the levels of treatment satisfaction or dissatisfaction between fingolimod and dimethyl fumarate with regard to effectiveness, side effects and convenience and to describe other patient-reported outcomes related to social impact and disability.

## Methods

This was a cross-sectional observational study in which patients with RRMS in the United States with current or past experience of fingolimod or dimethyl fumarate completed a web-based survey.

### Survey development

A draft online survey comprising a battery of measures was developed. The first section included questions about nine medication-specific side effects representing the top five side effects that could be perceived by patients for each of the two products based on incidence versus placebo as reported in the FDA product labels for each product (fingolimod: back pain, cough, diarrhea, headache, influenza; dimethyl fumarate: abdominal pain, diarrhea, flushing, nausea, vomiting) [7, 9]. The labelled side effect “influenza” was asked of patients in the survey as “flu-like symptoms.” All participants were presented with the same set of nine side effects.

The treatment satisfaction questionnaire for medication (TSQM), a 14-item scale measuring patient satisfaction with medication, including side effects, effectiveness, convenience, and global satisfaction [13], was administered. All TSQM scores range from 0 to 100, of which higher scores indicate greater treatment satisfaction.

A single item was developed to assess the impact of MS on social activities over the past 4 weeks, with five verbal response options (‘none of the time’ to ‘all of the time’).

Disability status was assessed using the multiple sclerosis rating scale-revised (MSRS-R), a patient-reported measure of MS-related functional status that has been validated psychometrically [14] and clinically in a population of patients with RRMS [15]. The scale covers the functional domains of “Walking,” “Using arms and hands,” “Vision,” “Speaking clearly,” “Swallowing,” “Bowel or bladder dysfunction,” “Thinking/Cognition/Memory,” and “Numbness/Tingling/Burning Sensation/Pain.” Scores for each scale range from 0 to 4, and the Total MSRS-R score ranges from 0 to 32, of which higher scores indicate greater disability. Disability was assessed using the patient-determined disease steps (PDDS) questionnaire [16], a 9-level scale providing a score indicating the patient-reported level of overall disability, ranging from 0 (i.e. normal walking) to 9 (i.e. bedridden).

The draft survey underwent pilot testing in which 5 patients completed the survey online and participated in an interview to provide feedback on the clarity and appropriateness of instructions, items and response options. Based on feedback, minor changes in the wording of items were made to improve clarity or to maintain consistency across similar items, and additional response options were added to some items to fully capture possible options.

### Study population

Study participants were recruited from an online patient community, PatientsLikeMe, a patient-powered research network that allows patients with MS to interact, track their health data over time, contribute to research [15], and improve their self-management [17]. All participants in the online community reporting that they had been diagnosed with MS by a physician were invited via email and provided with a URL to the online screener. Eligibility criteria included reported diagnosis of RRMS, current or past experience of treatment with fingolimod or dimethyl fumarate, aged 18 or older, and fluency in English. Any patient who reported they were enrolled in a clinical trial was also excluded from participation. Prior treatment with other DMTs was permitted. If they met the eligibility criteria, participants were presented with an online informed consent form, and only those who consented proceeded to the online survey. In order to have sufficient experience with either medication, participants must have been on either treatment for a minimum of 7 days so that patients would have had time to experience the product and, for those on fingolimod, to have completed the early monitoring phase; anyone who had initiated treatment less than 7 days prior to screening was asked to wait to complete the survey until they had been on treatment for at least 7 days. In this case, the participant was sent a reminder email after the 8th day of treatment to complete the survey. The study protocol was approved by New England IRB (Newton, MA). Participants were not remunerated for taking part in the study; at the end of the study all participants who completed the survey were emailed a brief report summarizing the findings.

### Data analysis

The primary analyses included all eligible participants who responded to the survey according to the following defined four study groups: participants currently receiving fingolimod (CF), participants currently receiving dimethyl fumarate (CDMF), participants who started and then discontinued fingolimod (DF), and those who started and then discontinued dimethyl fumarate (DDMF). Any patient who fell into more than one group based on treatment experience was assigned to a group based on his or her most recent treatment experience. For example, if a patient discontinued fingolimod and dimethyl fumarate, the patient was assigned to a group based on the treatment the patient was on most recently.

For each study group (i.e., CF, CDMF, DF, DDMF), descriptive statistics, including mean, standard deviation, and range for continuous variables, and frequency for categorical variables, were generated for medication-specific side effects of interest, the four scales of the TSQM, the single social functioning item, the PDDS questionnaire, the MSRS-R and for demographic and clinical characteristics. Descriptive statistics were generated for patients in the DF and DDMF groups to summarize characteristics related to therapy discontinuation.

Logistic regression models were employed to determine the association between study group (CF vs. CDMF) and the occurrence of side effects (Yes/No). Due to the small number of events observed for some side effects, a modification to the logistic regression models was implemented in an attempt to minimize potential bias due to quasi-complete separation of data. Hence, the Firth penalized-likelihood approach was used in all logistic regression models so that the issue of small sample sizes and separation of data points was addressed in the analysis [18]. For each side effect, two logistic regression models were generated: a univariable model and a multivariable model that adjusted for age, gender, and Total MSRS-R score. Age and Total MSRS-R score were included as continuous variables, while gender (male vs. female) was included as a categorical variable. Additionally, the occurrence of any side effect (i.e., the indication of any of the listed side effects) was also modeled as one of the outcomes.

An analysis of variance (ANOVA) model and an analysis of covariance (ANCOVA) model were utilized to determine the association between study groups (CF vs. CDMF) and each scale of the TSQM score. In the ANOVA model, the only independent variable was study group (CF or CDMF). In the ANCOVA model, the independent variables were the same as in the multivariable logistic regression models. For each study group the least-squares mean was presented along with the 95 % confidence interval and *p* value. These same statistics were also presented for the difference between study groups, and this difference represented the main measure of association of study group effect. Because the objective of this study was not to prove or disprove a specific hypothesis, no threshold alpha value was set upon which to make a decision of significance. Due to the exploratory nature of these analyses, all *p* values were interpreted as descriptive statistics. Sponsorship for this study and article processing charges was funded by Novartis Pharmaceuticals Corporation.

## Results

The survey was fielded from 3 April 2014 to 8 August 2014. A total of 6256 patients were invited, 470 were screened as eligible, 206 were screened as ineligible (per inclusion and exclusion criteria) and a total of 283 completed the survey. Two patients were excluded from the analysis because their data indicated they had initiated current therapy less than 7 days prior to survey completion. The final sample sizes for the 281 eligible survey completers were as follows: CF (N = 61), CDMF (N = 129), DF (N = 32), DDMF (N = 59). Demographic and clinical characteristics for the study sample are presented in Table 1. Overall, observed underlying demographic and clinical characteristics were generally similar across all study groups; however, the sample was generally overrepresented by the female gender, especially in the DF and DDMF subgroups.

**Table 1:** Sociodemographic and clinical characteristics

Characteristic	CF (N = 61)	CDMF (N = 129)	DF (N = 32)	DDMF (N = 59)
Gender, n (%)				
Male	14 (23.0 %)	26 (20.2 %)	2 (6.3 %)	8 (13.6 %)
Female	47 (77.0 %)	103 (79.8 %)	30 (93.8 %)	51 (86.4 %)
Age (years)				
Mean (SD)	46.2 (10.5)	50.4 (9.7)	47.6 (9.5)	51.8 (10.1)
Min, max	24.0, 65.0	27.0, 72.0	27.0, 66.0	29.0, 72.0
Ethnicity, n (%)				
Hispanic	1 (1.6 %)	1 (0.8 %)	1 (3.1 %)	2 (3.4 %)
Not hispanic	58 (95.1 %)	124 (96.1 %)	29 (90.6 %)	54 (91.5 %)
Prefer not to answer	2 (3.3 %)	4 (3.1 %)	2 (6.3 %)	3 (5.1 %)
Race, n (%)				
White	53 (86.9 %)	113 (87.6 %)	27 (84.4 %)	53 (89.8 %)
Black	5 (8.2 %)	8 (6.2 %)	1 (3.1 %)	2 (3.4 %)
Asian	0 (0.0 %)	1 (0.8 %)	1 (3.1 %)	0 (0.0 %)
Native American/Alaska Native	0 (0.0 %)	0 (0.0 %)	0 (0.0 %)	1 (1.7 %)
Mixed race	3 (4.9 %)	3 (2.3 %)	2 (6.3 %)	2 (3.4 %)
Prefer not to answer	0 (0.0 %)	4 (3.1 %)	1 (3.1 %)	1 (1.7 %)
Disease duration (years)				
Mean (SD)	9.8 (7.7)	11.0 (8.3)	14.8 (9.5)	13.0 (9.6)
Min, max	0.3, 35.5	0.1, 35.9	2.2, 39.6	0.3, 48.8
Disability according to patient-determined disease steps (PDDS), n (%)				
Normal	16 (26.2 %)	17 (13.2 %)	8 (25.0 %)	4 (6.8 %)
Mild disability	8 (13.1 %)	21 (16.3 %)	4 (12.5 %)	1 (1.7 %)
Moderate disability	8 (13.1 %)	12 (9.3 %)	3 (9.4 %)	10 (16.9 %)
Gait disability	16 (26.2 %)	20 (15.5 %)	4 (12.5 %)	12 (20.3 %)
Early cane	4 (6.6 %)	21 (16.3 %)	2 (6.3 %)	12 (20.3 %)
Late cane	5 (8.2 %)	21 (16.3 %)	5 (15.6 %)	10 (16.9 %)
Bilateral support	2 (3.3 %)	7 (5.4 %)	4 (12.5 %)	6 (10.2 %)
Wheelchair/scooter	2 (3.3 %)	10 (7.8 %)	2 (6.3 %)	4 (6.8 %)
During the past 4 weeks how much has your physical health interfered with your social activities?, n (%)				
None of the time	11 (18.0 %)	18 (14.0 %)	3 (9.4 %)	3 (5.1 %)
A little of the time	18 (29.5 %)	32 (24.8 %)	9 (28.1 %)	10 (16.9 %)
Some of the time	22 (36.1 %)	46 (35.7 %)	7 (21.9 %)	15 (25.4 %)
Most of the time	6 (9.8 %)	23 (17.8 %)	9 (28.1 %)	25 (42.4 %)
All of the time	4 (6.6 %)	10 (7.8 %)	4 (12.5 %)	6 (10.2 %)
Education level, n (%)				
Some high school	0 (0.0 %)	1 (0.8 %)	0 (0.0 %)	0 (0.0 %)
High school grad or GED	5 (8.2 %)	12 (9.3 %)	3 (9.4 %)	8 (13.6 %)
Some college	26 (42.6 %)	64 (49.6 %)	11 (34.4 %)	25 (42.4 %)
Undergraduate degree	15 (24.6 %)	36 (27.9 %)	8 (25.0 %)	13 (22.0 %)
Postgraduate degree	15 (24.6 %)	15 (11.6 %)	10 (31.3 %)	12 (20.3 %)
Prefer not to answer	0 (0.0 %)	1 (0.8 %)	0 (0.0 %)	1 (1.7 %)
Household annual income, n (%)				
Prefer not to say	10 (16.4 %)	30 (23.3 %)	9 (28.1 %)	12 (20.3 %)
Less than $24,999	10 (16.4 %)	25 (19.4 %)	4 (12.5 %)	10 (16.9 %)
$25,000–39,999	8 (13.1 %)	15 (11.6 %)	4 (12.5 %)	6 (10.2 %)
$40,000–79,999	16 (26.2 %)	27 (20.9 %)	4 (12.5 %)	18 (30.5 %)
80,000 to $119,999	7 (11.5 %)	17 (13.2 %)	5 (15.6 %)	6 (10.2 %)
$120,000 or more	10 (16.4 %)	15 (11.6 %)	6 (18.8 %)	7 (11.9 %)

Across both current therapy groups, the most commonly reported MS medication taken before initiating current therapy was Copaxone (glatiramer acetate) (see Table 2). CF patients were more likely to have taken Copaxone as their most recent prior therapy (CF: 27.9 %, CDMF: 19.4 %), while CDMF patients were more likely to have taken a break from treatment before starting dimethyl fumarate (CDMF: 9.3 %, CF: 1.6 %,) or to have taken Rebif^®^ (interferon beta-1a) as their most recent prior therapy (CDMF: 18.6 %, CF: 8.2 %). Side effects of prior medication were cited by 67.3 % of CF and 44.1 % of CDMF patients as a reason for switching to current therapy. Other commonly reported reasons were: wanting to take oral treatment (CF: 63.3 %, CDMF: 61.8 %), perceived lack of effectiveness of previous medication (CF: 38.8 %, CDMF: 31.4 %), struggling to take previous medication as prescribed (CF: 32.7 %, CDMF: 15.7 %), and concerns regarding safety of previous medication (CF: 28.6 %, CDMF: 27.5 %).

**Table 2:** Characteristics of patients in the current fingolimod (CF) and current dimethyl fumarate (CDMF) groups

Characteristic, n (%)	CF (N = 61)	CDMF (N = 129)
MS medication before taking this therapy		
Copaxone (glatiramer acetate)	17 (27.9 %)	25 (19.4 %)
Tysabri (natalizumab)	9 (14.8 %)	21 (16.3 %)
No other med, this was first drug	9 (14.8 %)	14 (10.9 %)
Tecfidera or Gilenya (Dimethyl Fumerate)	6 (9.8 %)	14 (10.9 %)
Avonex (interferon beta1a)	6 (9.8 %)	11 (8.5 %)
Rebif (interferon beta 1a)	5 (8.2 %)	24 (18.6 %)
Betaseron (interferon beta1b)	5 (8.2 %)	4 (3.1 %)
Other medicine not listed	2 (3.3 %)	1 (0.8 %)
No other med, took break from treatment	1 (1.6 %)	12 (9.3 %)
Aubagio (teriflunomide)	1 (1.6 %)	2 (1.6 %)
Extavia (interferon beta 1b)	0 (0.0 %)	1 (0.8 %)
Reasons switched to current therapy		
Previous medication side effects		
Yes	33 (67.3 %)	45 (44.1 %)
No	16 (32.7 %)	57 (55.9 %)
Wanted to take oral treatment		
Yes	31 (63.3 %)	63 (61.8 %)
No	18 (36.7 %)	39 (38.2 %)
Previous medication lack of effectiveness		
Yes	19 (38.8 %)	32 (31.4 %)
No	30 (61.2 %)	70 (68.6 %)
Struggled to take previous medication as prescribed		
Yes	16 (32.7 %)	16 (15.7 %)
No	33 (67.3 %)	86 (84.3 %)
Concerns regarding safety of previous medication		
Yes	14 (28.6 %)	28 (27.5 %)
No	35 (71.4 %)	74 (72.5 %)
Another reason not listed		
Yes	9 (18.4 %)	25 (24.5 %)
No	40 (81.6 %)	77 (75.5 %)
Change in health status		
Yes	8 (16.3 %)	24 (23.5 %)
No	41 (83.7 %)	78 (76.5 %)
Financial reasons		
Yes	2 (4.1 %)	4 (3.9 %)
No	47 (95.9 %)	98 (96.1 %)

Among the discontinued groups, DDMF patients were more likely than DF to indicate side effects as one of the reasons for discontinuing therapy (78.0 vs. 43.8 %, respectively) (see Table 3). When asked to provide the main reason for discontinuation, the three most common responses were side effects (DF: 46.9 %, DDMF: 67.8 %), lack of effectiveness (DF: 25.0 %, DDMF: 15.3 %), and another reason not listed (DF: 15.6 %, DDMF: 10.2 %). Approximately half of the patients in each group indicated that the decision to discontinue therapy was a joint decision between the respondent and his or her doctor. A slightly greater percentage of DDMF patients (40.7 %) reported that the decision was mainly theirs compared to DF patients (28.1 %), but the overall distribution of responses between groups was similar.

**Table 3:** Characteristics of discontinued users

Characteristic, n (%)	DF (N = 32)	DDMF (N = 59)
When did you start therapy?, n (%)		
Within the last 7 days	0 (0.0 %)	1 (1.7 %)
Between 8 and 30 days ago	0 (0.0 %)	5 (8.5 %)
Between 31 and 60 days ago	1 (3.1 %)	2 (3.4 %)
More than 60 days ago	31 (96.9 %)	51 (86.4 %)
When did you stop therapy?, n (%)		
Within the last 7 days	0 (0.0 %)	2 (3.4 %)
Between 8 and 30 days ago	1 (3.1 %)	14 (23.7 %)
Between 31 and 60 days ago	3 (9.4 %)	8 (13.6 %)
More than 60 days ago	28 (87.5 %)	35 (59.3 %)
Reasons stopped taking therapy^a^, n (%)		
Side effects	14 (43.8 %)	46 (78.0 %)
Doctor advice	8 (25.0 %)	14 (23.7 %)
Lack of effectiveness in treating MS	8 (25.0 %)	13 (22.0 %)
Concerns regarding the safety	5 (15.6 %)	5 (8.5 %)
Struggled to take therapy as prescribed	1 (3.1 %)	3 (5.1 %)
Financial reasons	1 (3.1 %)	2 (3.4 %)
Did not want to take oral treatment	0 (0.0 %)	1 (1.7 %)
Changes to health plan benefits	1 (3.1 %)	0 (0.0 %)
Another reason not listed	6 (18.8 %)	7 (11.9 %)
Main reason stopped taking therapy, n (%)		
Side effects	15 (46.9 %)	40 (67.8 %)
Lack of effectiveness in treating MS	8 (25.0 %)	9 (15.3 %)
Doctor advice	2 (6.3 %)	3 (5.1 %)
Concerns regarding safety	2 (6.3 %)	0 (0.0 %)
Financial reasons	0 (0.0 %)	1 (1.7 %)
Another reason not listed	5 (15.6 %)	6 (10.2 %)
Who mainly made decision to discontinue therapy?, n (%)		
Mainly my decision	9 (28.1 %)	24 (40.7 %)
Mainly doctor decision	7 (21.9 %)	8 (13.6 %)
Joint decision	16 (50.0 %)	27 (45.8 %)

^a^Participants could select more than one response

### Tolerability

Among the current therapy groups, CDMF patients were more likely than CF patients to report abdominal pain, diarrhea, flushing, flu-like symptoms, and nausea as treatment side effects (see Table 4). The reported occurrence of the remaining side effects was similar between the two groups. Logistic regression model results summarizing the treatment effect in predicting the occurrence of each side effect are presented in Table 5. After adjusting for covariates, CDMF patients had an increased risk of experiencing abdominal pain (odds ratio, i.e. OR 15.79), flushing (OR 12.51), and any of the solicited side effects (OR 7.49). Results from the univariable model and the multivariable model yielded similar results. Other side effects for which a pronounced increase in risk was observed for CDMF patients after adjustment were diarrhea (OR 2.30) and nausea (OR 3.16).

**Table 4:** Frequency and severity of side effects among current users

Side effect	Ever experienced while on current medication, N (%)		Experienced in last 7 days, N (%)		Mean number of days experienced in last 7 days, mean (SD)		Mean severity rating in last 7 days, mean (SD) (1–10 scale)^a^	
	CF (N = 61)	CDMF (N = 129)	Current fingolimod (CF) ever experienced side effect while on current medication	Current dimethyl fumarate (CDMF) ever experienced side effect while on current medication	Current fingolimod (CF) experienced side effect in last 7 days	Current dimethyl fumarate (CDMF) experienced side effect in last 7 days	Current fingolimod (CF) experienced side effect in last 7 days	Current dimethyl fumarate (CDMF) experienced side effect in last 7 days
Abdominal pain	2 (3.3 %)	48 (37.2 %)	1/2 (50.0 %)	18/48 (37.5 %)	7.0 (NA) n = 1	3.3 (2.1) n = 18	4.0 (NA) n = 1	4.8 (2.0) n = 18
Back pain	5 (8.2 %)	11 (8.5 %)	2/5(40.0 %)	4/11 (36.4 %)	5.0 (2.8) n = 2	6.5 (1.0) n = 4	5.0 (0.0) n = 2	5.8 (2.8) n = 4
Cough	6 (9.8 %)	17 (13.2 %)	3/6 (50.0 %)	11/17 (64.7 %)	5.7 (1.2) n = 3	4.9 (2.3) n = 11	3.3 (2.1) n = 3	4.3 (1.8) n = 11
Diarrhea	10 (16.4 %)	40 (31.0 %)	4/10 (40.0 %)	16/40 (40.0 %)	4.3 (2.2) n = 4	3.8 (1.9) n = 16	5.8 (1.9) n = 4	5.6 (2.3) n = 16
Flushing	12 (19.7 %)	99 (76.7 %)	6/12 (50.0 %)	54/99 (54.5 %)	4.7 (2.6) n = 6	4.1 (2.3) n = 54	5.2 (1.7) n = 6	5.0 (2.9) n = 54
Headache	18 (29.5 %)	34 (26.4 %)	9/18 (50.0 %)	14/34 (41.2 %)	4.4 (2.1) n = 9	4.5 (2.1) n = 14	5.3 (2.9) n = 9	5.1 (2.6) n = 14
Flu-like symptoms	2 (3.3 %)	15 (11.6 %)	1/2 (50.0 %)	5/15 (33.3 %)	5.0 (NA) n = 1	2.2 (1.3) n = 5	6.0 (NA) n = 1	3.2 (3.2) n = 5
Nausea	8 (13.1 %)	44 (34.1 %)	1/8 (12.5 %)	22/44 (50.0 %)	4.0 (NA) n = 1	2.9 (1.5) n = 22	8.0 (NA) n = 1	4.4 (2.0) n = 1
Vomiting	4 (6.6 %)	11 (8.5 %)	1/4 (25.0 %)	2/11 (18.2 %)	2.0 (NA) n = 1	1.5 (0.7) n = 2	6.0 (NA) n = 1	7.5 (0.7) n = 2

^a^Severity rated on a 1–10 scale with 1 being very mild and 10 as bad as one can imagine

**Table 5:** Side effects: logistic regression analyses

Side effect	Univariable results			Multivariable results^a^		
	Odds ratio CDMF vs CF	95 % CI	p value	Odds ratio CDMF vs CF	95 % CI	p value
Any side effect	7.11	3.45, 14.62	<0.001	7.49	3.54, 15.86	<0.001
Abdominal pain	14.16	3.77, 53.27	<0.001	15.79	4.20, 59.41	<0.001
Flushing	12.92	6.12, 27.25	<0.001	12.51	5.85, 26.76	<0.001
Nausea	3.28	1.45, 7.40	0.004	3.16	1.38, 7.24	0.007
Flu-like symptoms	3.22	0.81, 12.84	0.097	3.02	0.77, 11.82	0.112
Diarrhea	2.22	1.03, 4.77	0.041	2.30	1.06, 5.00	0.035
Cough	1.33	0.51, 3.48	0.563	1.32	0.50, 3.44	0.574
Vomiting	1.24	0.40, 3.89	0.712	1.26	0.41, 3.91	0.683
Back pain	1.00	0.34, 2.91	0.995	0.94	0.33, 2.72	0.915
Headache	0.85	0.43, 1.67	0.635	0.87	0.43, 1.73	0.687

^a^Covariates: age (continuous), gender (male/female), and Total MSRS-R score (continuous)

Among patients in the discontinued therapy groups, a group effect was observed with DDMF patients more than twice as likely to experience the following side effects (DDMF vs. DF): abdominal pain (57.6 vs. 15.6 %), diarrhea (44.1 vs. 15.6 %), flushing (72.9 vs. 25.0 %), nausea (52.2 vs. 18.8 %), and vomiting (28.8 vs. 6.3 %). The reported occurrence of the remaining side effects were more similar between discontinued therapy groups (DDMF vs. DF): back pain (20.3 vs 12.5 %), cough (10.2 vs. 12.5 %), headache (44.1 vs. 31.3 %), and flu-like symptoms (27.1 vs. 21.9 %).

### Treatment satisfaction

ANCOVA analyses modeling TSQM scores as a function of study group (CF vs. CDMF) are presented in Table 6. CF patients on average had better convenience scale scores (8.1 points higher than CDMF patients) and better side effects scale scores (9.5 points higher than CDMF patients). There was no difference between the two groups on the “Effectiveness” scale. Differences between the groups in the global score indicate a marginal treatment effect related to overall treatment satisfaction favoring the CF group.

**Table 6:** TSQM domain and global scores: ANCOVA analyses

TSQM scale	ANOVA			ANCOVA^a^		
	LS mean^b^	95 % CI	p value^c^	LS mean^b^	95 % CI	p value^c^
Effectiveness						
CF	65.2	59.9, 70.5		65.0	59.6, 70.5	
CDMF	66.1	62.5, 69.8		66.2	62.5, 70.0	
Difference	−0.9	−7.4, 5.5	0.776	−1.2	−7.9, 5.4	0.720
Convenience						
CF	94.4	90.6, 98.3		94.8	90.9, 98.6	
CDMF	86.8	84.2, 89.4		86.7	84.1, 89.3	
Difference	7.6	3.0, 12.3	0.001	8.1	3.4, 12.8	<0.001
Side effects						
CF	91.6	85.6, 97.5		91.3	85.3, 97.3	
CDMF	81.6	77.8, 85.5		81.8	77.9, 85.6	
Difference	10.0	2.8, 17.1	0.007	9.5	2.3, 16.8	0.010
Global						
CF	76.0	70.2, 81.8		75.5	69.7, 81.4	
CDMF	68.8	64.8, 72.8		69.0	65.0, 73.0	
Difference	7.2	0.1, 14.2	0.047	6.5	−0.6, 13.7	0.076

^a^Covariates: age (continuous), gender (male/female), and Total MSRS-R score (continuous)

^b^Higher scores indicate higher treatment satisfaction (greater perceived effectiveness, greater satisfaction, fewer/less severe side effects, greater global satisfaction)

^c^p values are provided as descriptive statistics to assess the degree of difference between each LS mean estimate and zero

As expected, treatment satisfaction scores for the discontinued therapy groups were lower than the scores for the current therapy groups across all TSQM scales. However, DF patients tended to have higher mean treatment satisfaction scores than DDMF patient across all TSQM scales: effectiveness (DF: 41.7, SD = 27.3 vs. DDMF: 27.3, SD = 20.4), convenience (DF: 88.9, SD = 13.5 vs. DDMF: 79.1, SD = 20.8), side effects (DF: 81.0, SD = 36.5 vs. DDMF: 49.5, SD = 39.2), global score (DF: 28.3, SD = 25.1 vs. DDMF: 14.4, SD = 17.5) (results not tabulated).

## Discussion

This study provides real-world evidence of the tolerability and overall treatment satisfaction among patients with RRMS using fingolimod and dimethyl fumarate. As seen in randomized, controlled trials and clinical studies, side effects associated with dimethyl fumarate (i.e., abdominal pain, diarrhea, flushing, nausea, vomiting) were more commonly experienced by those in the dimethyl fumarate group, supporting other reports of the side effect profile of this treatment [11]. However, the frequency of side effects associated with fingolimod (i.e., back pain, cough, diarrhea, headache, influenza) was comparable between groups. Among study participants, the odds ratio of experiencing a side effect was approximately seven, in favor of fingolimod over dimethyl fumarate. Associations remained strong after multivariable adjustment for age and disability, including after a second multivariable model adjusting for PDDS instead of MSRS-R as the measure of disability.

For both treatment groups, discontinuation was commonly driven by the experience of side effects. This was particularly evident for dimethyl fumarate, with 68 % of patients reporting “experience of side effects” as the main reason for discontinuation, and more patients in this group deciding unilaterally to stop treatment. Discontinuation of fingolimod was more frequently reported to result from a lack of perceived efficacy (25 %) than dimethyl fumarate (15 %). Compared to patients taking dimethyl fumarate, those currently taking fingolimod were more satisfied with treatment overall and specifically with regard to side effects and convenience. This was also true among the discontinued patients, with those who had discontinued fingolimod reporting greater treatment satisfaction than those who had discontinued dimethyl fumarate. However, perception of treatment effectiveness was similar between the dimethyl fumarate and fingolimod groups and use of the former drug appeared to be twice as frequent in this sample. After adjusting for underlying group characteristics, the similar perceived efficacy remained the same.

Studies in diseases involving long-term treatments have shown an association between side effects and poor compliance [19–21]. Additionally, studies among MS patients have shown that increased adherence leads to better patient outcomes and reduced healthcare utilization [5, 22, 23]. Thus, DMTs with better tolerability have the potential to improve outcomes and reduce resource use for RRMS patients, as a result of increased adherence.

Despite the consistency of results, there are several limitations of the study that should be noted. The small sample sizes did not allow for the detection of potential trends between the treatment groups. Although other oral DMTs such as teriflunomide (Aubagio^®^, Biogen) have recently been approved for use in RRMS, this study was limited to the two DMTs most commonly used at the time on PatientsLikeMe. Because accurate data were not publicly available to describe the true demographics of this subset of the MS population, it was not possible to use benchmark weighting to try to control for such differences. Additionally, many patients had started therapy several months before completing the survey, and given that all data was patient-reported, there is the potential for recall error regarding medication use, the side effects experienced or reasons for discontinuation. Those who agreed to participate might not be representative of the population of RRMS patients at large, who may not use internet, health-sharing sites, nor complete online surveys. Finally, the cross-sectional study design limited the ability to ascertain how the side effect experience may affect longer term adherence or prognostic outcomes.

In conclusion, the patient experience of fingolimod and dimethyl fumarate was similar for some measures and favored fingolimod for others. Future research involving a larger sample and a longitudinal design would be useful to provide further insights on real-world patient experiences and to examine the relationship between tolerability and adherence/persistence.

## Abbreviations

ANCOVA: analysis of covariance
ANOVA: analysis of variance
CDMF: currently receiving dimethyl fumarate
CF: currently receiving fingolimod
DDMF: discontinued dimethyl fumarate
DF: discontinued fingolimod
DMT: disease-modifying therapies
FDA: Food and Drug Administration
MS: multiple sclerosis
MSRS-R: multiple sclerosis rating scale-revised
OR: odds ratio
PDDS: patient-determined disease steps questionnaire
RRMS: relapsing-remitting multiple sclerosis
SD: standard deviation
TSQM: treatment satisfaction questionnaire for medication

## References

[1] Tullman MJ (2013). Overview of the epidemiology, diagnosis, and disease progression associated with multiple sclerosis. Am J Manag Care.

[2] Bergvall N, Petrilla AA, Karkare SU, Lahoz R, Agashivala N, Pradhan A (2014). Persistence with and adherence to fingolimod compared with other disease-modifying therapies for the treatment of multiple sclerosis: a retrospective US claims database analysis. J Med Econ.

[3] Menzin J, Caon C, Nichols C, White LA, Friedman M, Pill MW (2013). Narrative review of the literature on adherence to disease-modifying therapies among patients with multiple sclerosis. J Manag Care Pharm.

[4] Devonshire V, Lapierre Y, Macdonnel R, Ramo-Tello C, Patti F, Fontoura P (2011). The Global Adherence Project (GAP): a multicenter observational study on adherence to disease-modifying therapies in patients with relapsing-remitting multiple sclerosis. Eur J Neurol.

[5] Agashivala N, Wu N, Abouzaid S, Wu Y, Kim E, Boulanger L (2013). Compliance to fingolimod and other disease modifying treatments in multiple sclerosis patients, a retrospective study. BMC Neurol.

[6] Wicks P, Massagli M, Kulkarni A, Dastani H (2011). Online development of a tool to measure barriers to adherence: the multiple sclerosis treatment adherence questionnaire (MS-TAQ). J Med Internet Res.

[7] Gilenya [package insert]. Stein (CH): Novartis Pharma Stein AG; 2012.

[8] Cohen JA, Barkhof F, Comi G, Hartung HP, Khatri BO, Montalban X (2010). TRANSFORMS study group. Oral fingolimod or intramuscular interferon for relapsing multiple sclerosis. N Engl J Med.

[9] Tecfidera. Cambridge (MA): Biogen Inc; 2013.

[10] Viglietta V, Miller D, Bar-Or A, Phillips JT, Arnold DL, Selmaj K (2015). Efficacy of delayed-release dimethyl fumarate in relapsing-remitting multiple sclerosis: integrated analysis of the phase 3 trials. Ann Clin Transl Neurol.

[11] Phillips JT, Hutchinson M, Fox R, Gold R, Havrdova E (2014). Managing flushing and gastrointestinal events associated with delayed-release dimethyl fumarate: experiences of an international panel. Mult Scler Relat Disord.

[12] Ontaneda D, Hara-Cleaver C, Rudick RA, Cohen JA, Bermel RA (2012). Early tolerability and safety of fingolimod in clinical practice. J Neurol Sci.

[13] Atkinson MJ, Sinha A, Hass SL, Colman SS, Kumar RN, Brod M (2004). Validation of a general measure of treatment satisfaction, the treatment satisfaction questionnaire for medication (TSQM), using a national panel study of chronic disease. Health Qual Life Outcomes.

[14] Wicks P, Vaughan TE, Massagli MP (2012). The multiple sclerosis rating scale, revised (MSRS-R): development, refinement, and psychometric validation using an online community. Health Qual Life Outcomes.

[15] Bove R, Secor E, Healy BC, Musallam A, Vaughan T, Glanz BI (2013). Evaluation of an online platform for multiple sclerosis research: patient description, validation of severity scale, and exploration of BMI effects on disease progression. PLoS ONE.

[16] Hohol MJ, Orav EJ, Weiner HL (1995). Disease steps in multiple sclerosis: a simple approach to evaluate disease progression. Neurology.

[17] Wicks P, Massagli M, Frost J, Brownstein C, Okun S, Vaughan T (2010). Sharing health data for better outcomes on PatientsLikeMe. J Med Internet Res.

[18] Heinze G, Schemper M (2002). A Solution to the problem of separation in logistic regression. Stat Med.

[19] Braithwaite RS, Kozal MJ, Chang CC, Roberts MS, Fultz SL, Goetz MB (2007). Adherence, virological and immunological outcomes for HIV-infected veterans starting combination antiretroviral therapies. AIDS.

[20] Brod M, Rousculp M, Cameron A (2008). Understanding compliance issues for daily self-injectable treatment in ambulatory care settings. Patient Prefer Adherence.

[21] Conte P, Guarneri V (2004). Safety of intravenous and oral bisphosphonates and compliance with dosing regimens. Oncologist.

[22] Tan H, Cai Q, Agarwal S, Stephenson JJ, Kamat S (2011). Impact of adherence to disease-modifying therapies on clinical and economic outcomes among patients with multiple sclerosis. Adv Ther.

[23] Steinberg SC, Faris RJ, Chang CF, Chan A, Tankersley MA (2010). Impact of adherence to interferons in the treatment of multiple sclerosis: a non-experimental, retrospective, cohort study. Clin Drug Investig.

